# Relationship Between Tei Index and PEP-Derived Myocardial Performance Index in Sinus Rhythm

**DOI:** 10.1097/MD.0000000000001112

**Published:** 2015-07-24

**Authors:** Feyzullah Besli, Cengiz Basar, Ismail Ekinozu, Yasin Turker

**Affiliations:** From the Department of Cardiology (FB), Sanliurfa Mehmet Akif Inan Training and Research Hospital, Sanliurfa; Department of Cardiology (CB), Duzce Ataturk State Hospital; and Department of Cardiology (IE, YT), Duzce University Faculty of Medicine Hospital, Duzce, Turkey.

## Abstract

The goal of this study was to evaluate the preejection time (PEP)-derived myocardial performance index (MPI) in hypertensive (HT) patients with sinus rhythm and its relationship to the classic Tei index.

One hundred five patients were enrolled in the study (65 HT and 40 control subjects). The mean age of all patients was 50.5 ± 15 years and 60% were female. Echocardiography was performed on all patients. MPI was measured with the classic Tei method (MPI-Tei index) and the PEP-derived MPI method by using tissue Doppler echocardiography. Although the MPI-Tei index is defined as the ratio of isovolumetric contraction time (IVCT) along with isovolumetric relaxation time (IVRT) to ejection time (ET), PEP-derived MPI is defined as the ratio of PEP and IVRT to ET. We compared echocardiographic data between the HT group and the control group.

MPI-Tei index and the PEP-derived MPI values were higher in the HT group compared with controls (0.52 ± 0.10 vs 0.39 ± 0.07, *P* < 0.001, and 0.51 ± 0.09 vs 0.39 ± 0.07). PEP-derived MPI was strongly correlated with the MPI-Tei index (*r* = 0.945, *P* < 0.001).

Our study determined that the PEP-derived MPI might be used in the evaluation of left ventricular function in patients with HT, similar to the classic MPI-Tei index.

## INTRODUCTION

The use of classical echocardiographic indexes has many limitations in the assessment of systolic and diastolic left ventricular (LV) function. Age, heart rate, cardiac conduction disturbances, and changes in loading affect the Doppler signal of transmitral flow, which is the most commonly used method for studying systolic and diastolic function. Chuwa Tei devised an index of myocardial performance (MPI or the Tei index) that evaluates the LV systolic and diastolic function in combination.^1^ The Tei index is calculated as the ratio of the sum of the isovolumetric contraction time (IVCT) and isovolumetric relaxation time (IVRT) over the ejection time (ET).^1,2^ The Tei index has proved to be a reliable method for the evaluation of LV systolic and diastolic performance, with clear advantages over older, established indexes.^3,4^ MPI values obtained from healthy subjects were 0.39 ± 0.05 for LV.^1^ The MPI has been demonstrated to be a powerful and independent prognostic indicator in patients with various cardiac disorders and has been studied in congestive heart failure syndrome, congenital heart diseases, cardiac rejection following transplantation, and valvular heart diseases.^4–7^

However, because of the loss of mechanical atrial function, the end of LV diastolic activity cannot be clearly determined in patients with atrial fibrillation (AF). Therefore, IVCT cannot be measured in patients with AF. Preejection period (PEP) can easily be obtained in AF patients, and PEP may be used instead of IVCT in AF patients.^8^ PEP interval was measured from the onset of QRS to the onset of the systolic mitral annular velocity pattern. Su et al^9^ defined the “PEPa-derived MPI” in patients with AF; it is calculated as the ratio of PEPa along with IVRTa to ETa. In a recent study, it was reported that the PEPa-derived MPI was a useful predictor of adverse cardiovascular events, and could offer an additional prognostic benefit over conventional clinical and echocardiographic parameters in patients with AF.

To our knowledge, the importance of PEP-derived MPI used in AF has not been determined in patients with sinus rhythm. Our goal was to evaluate the role of PEP-derived MPI in hypertensive (HT) patients with sinus rhythm and compare it to the Tei index.

## METHODS

### Study Design and Population

One hundred five patients who were admitted to the Duzce State Hospital and the Duzce University Department of Cardiology between March 2013 and September 2014 with HT (n = 65) and healthy subjects (n = 40) were included in the study. The mean age of all patients was 50.5 ± 15 years, and 60% were women. Informed consent was obtained from each subject for participation in the study. The study conforms to the principles outlined in the Declaration of Helsinki and was approved by the local Ethics Committee for clinical research. The clinical diagnosis of HT was made on the basis of clinical history and physical examination, and was defined as abnormally high arterial blood pressure (BP) that is usually indicated by an adult average systolic BP of ≥140 mm Hg or a diastolic BP of ≥90 mm Hg at rest.

Under the age of 18 years, rheumatic valvular disease or severe valvular disease, the history of valvular operations, diabetes mellitus, coronary artery disease, AF, cardiac conduction disturbances, severe pulmonary hypertension or chronic obstructive pulmonary disease, chronic infection or malignancy, and thyroid diseases were excluded from the study. Of the subjects enrolled in the study, age, sex, and medical history (duration of HT and medications) were questioned. The rhythms of patients at admission were identified by electrocardiogram (ECG). Following 12 hours of fasting, venous blood samples were collected for biochemical examinations. Echocardiography was performed in all the patients.

### Echocardiography

Two trained cardiologists performed the echocardiography and recorded images for each patient using the Vivid 3 model of the echocardiography device (General Electrics, Vivid 3 echocardiography, Milwaukee, WI). After the echocardiography was performed for all patients, raw echocardiographic images were analyzed offline with software (EchoPAC; GE Medical Systems, USA) by other 2 cardiologists blinded to the patients’ data. From the standard transthoracic windows, LV end-diastolic diameter (LVEDD), LV end-systolic diameter (LVESD), LV interventricular septum diameter (LV IVS), LV posterior wall (LV PW), and LV ejection fraction (EF) were measured. The Doppler sample volume was placed at the tips of the mitral leaflets to get the LV inflow waveforms from the apical 4-chamber view. All sample volumes were positioned with ultrasonic beam alignment to flow. Transmitral E wave velocity (E) and A wave velocity were obtained from the recorded data and were averaged to generate the mean value.^10^ Tissue Doppler imaging was obtained with the sample volume placed at the medial and lateral corner of the mitral annulus from the apical 4-chamber view. On the tissue Doppler images, IVCT, PEP, IVRT, ET, early-diastolic mitral annular velocity (Em), late-diastolic mitral annular velocity (Am), and peak systolic mitral annular velocity (S) were measured from the same cardiac cycles, and the data were averaged to give the mean value. The mean velocities on the tissue Doppler images were calculated by averaging the velocities from the 2 sites at 5 cardiac cycles from the recorded data.

### PEP-Derived MPI and Tei Index

**FIGURE 1:**
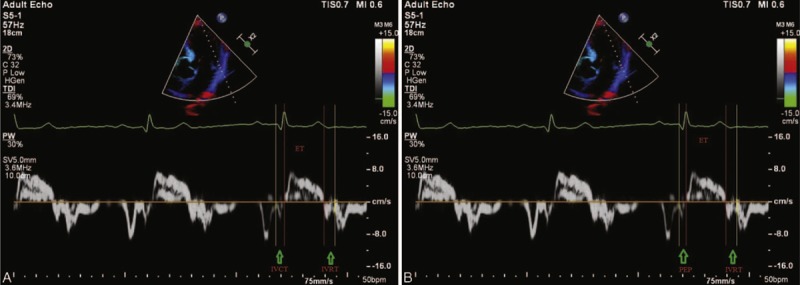
The measurment of PEP-derived myocardial performance index and myocardial performance index with Tei method, (A, B) Isovolumic contraction time (IVCT), preejection period (PEP), ejection time (ET), isovolumic relaxation time (IVRT), PEP-derived myocardial performance index (PEP-derived MPI), and myocardial performance index with Tei method (MPI-Tei index) obtained in a representative same hypertensive case. MPI-Tei index was defined as the ratio of IVCT + IVRT to ET, and PEP-derived MPI as the ratio of PEP + IVRT to ET. IVCT = 70, PEP = 58, IVRT = 102, and ET = 288 mseconds. MPI-Tei = 0.59 and PEPa-derived MPI = 0.55. MPI = myocardial performance index, MPI-Tei = myocardial performance index by using Tei method.

IVCT is defined as the interval measured from the end of the late-diastolic mitral annular velocity pattern to the onset of the systolic mitral annular velocity pattern; PEP is defined as the interval measured from the onset of QRS to the onset of the systolic mitral annular velocity pattern; ET is the interval measured from the onset to the end of the systolic mitral annular velocity pattern; and IVRT is the interval measured from the end of the systolic mitral annular velocity pattern to the onset of the diastolic mitral annular velocity pattern on the same cardiac cycle (Figure 1A and B).

The MPI, the Tei index, also called the MPI-Tei index, was defined as the ratio of IVCT along with IVRT to ET1, whereas the PEP-derived MPI was defined as the ratio of PEP along with IVRT to ET.^8^

### Reproducibility

The interobserver and intraobserver variability were calculated for 2 cardiologists who previously analyzed the recorded images offline. Fifteen patients were randomly selected to evaluate the interobserver variability of the PEP-derived MPI and the MPI-Tei index measurements by 2 independent observers. To determine the intraobserver variability, the same measurements were repeated 2 weeks apart. Mean percent error was calculated as the absolute difference divided by the average of the 2 observations.

### Statistical Analysis

The SPSS 15.0 (SPSS, Inc., Chicago, IL) was used for statistical analysis. The baseline, echocardiographic, and laboratory characteristics of study subjects are presented as percentages for dichotomous variables and mean ± standard deviation and as appropriate according to the distribution of the data. Categorical variables were given by number and percentage. The differences between groups were checked by χ^2^ test for categorical variables or by independent *t* test for continuous variables. The relationship between 2 continuous variables was assessed by a bivariate correlation method (Pearson correlation). All tests were 2 sided, and the level of significance was established as *P* < 0.05.

## RESULTS

The HT group was older than the controls (58.7 ± 11.3 vs 37.3 ± 12.1 years, *P* < 0.001). No significant differences in sex frequency, glucose, creatinine, white blood cells, hemoglobin, and platelet count were detected between the HT group and the controls (Table 1).

**TABLE 1:**
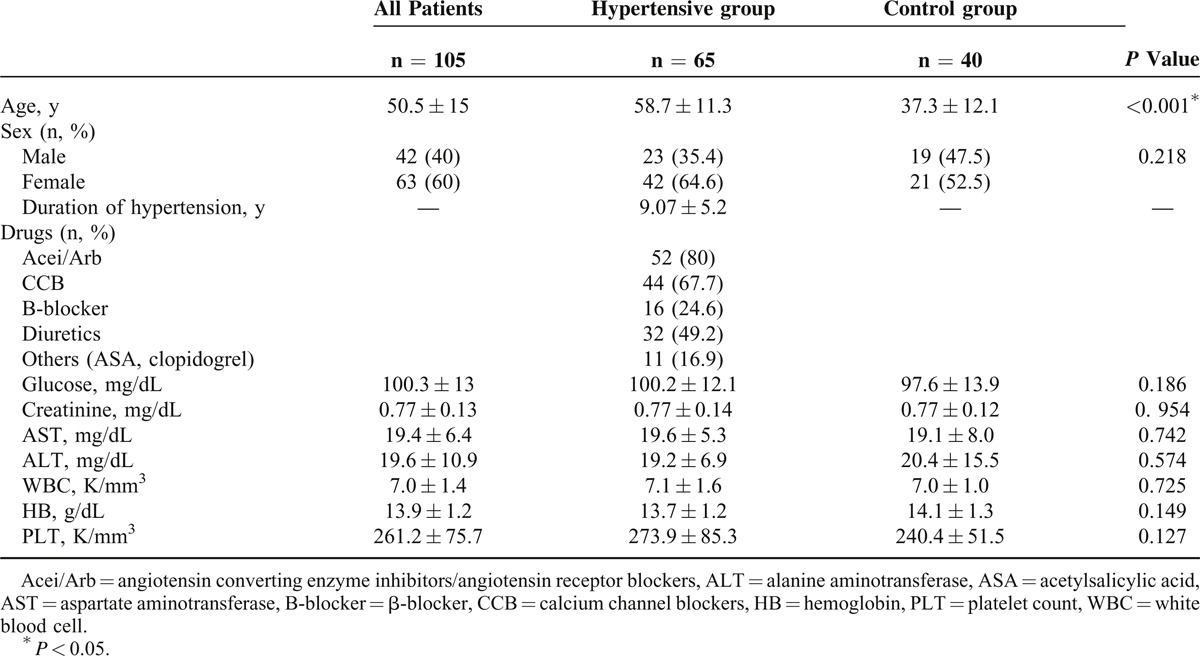
Baseline Clinical Characteristics and Laboratory Features of All Subjects, Hypertensive Group, and Control Group

When echocardiographic findings were evaluated, LV EF was 64.2 ± 4.3, MPI-Tei index was 0.47 ± 0.11, and PEP-derived MPI was 0.46 ± 0.10 in all patients. LV EF, LVDD, and LVSD exhibited no significant differences between the HT group and the control group. Compared with the control group, LV IVS (12.2 ± 1.5 vs 10.2 ± 0.9, *P* < 0.001), LV PW (11.3 ± 1.2 vs 10.0 ± 0.8, *P* < 0.001), A wave (93.5 ± 16.9 vs 77.1 ± 13.5, *P* < 0.001), Am (10.3 ± 2.1 vs 8.5 ± 2.3, *P* < 0.001), E/Em (9.5 ± 3.1 vs 7.1 ± 1.8, *P* < 0.001), IVCT (74.7 ± 22.3 vs 59.7 ± 21.2, *P* = 0.001), and PEP (70.5 ± 22.3 vs 60.2 ± 18.7, *P* = 0.009) were higher, whereas E wave (8.5 ± 2.5 vs 13.7 ± 3.4, *P* < 0.001), S (8.7 ± 1.8 vs 10.5 ± 1.8, *P* < 0.001), and Em (8.5 ± 2.5 vs 13.7 ± 3.4, *P* < 0.001) were lower in the HT group (Table 2).

**TABLE 2:**
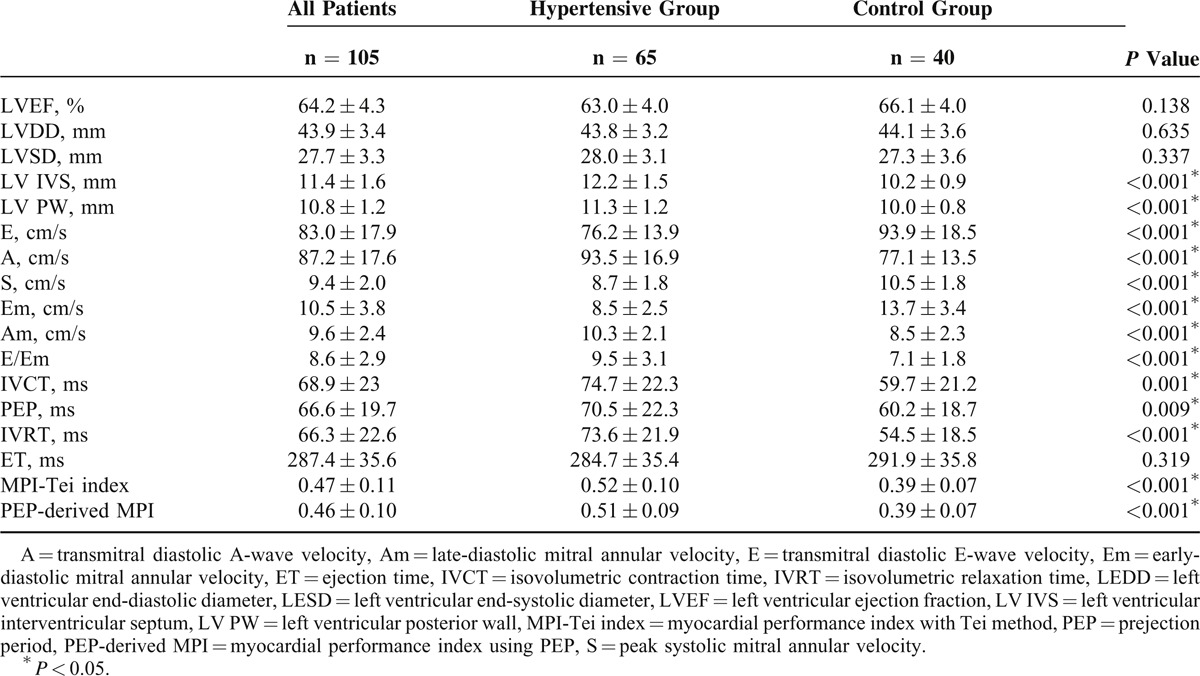
Echocardiographic Features of All Subjects, Hypertensive Group, and Control Group

Both the MPI-Tei index and the PEP-derived MPI values were higher in the HT group than controls (0.52 ± 0.10 vs 0.39 ± 0.07, *P* < .001, and 0.51 ± 0.09 vs 0.39 ± 0.07, *P* < 0.001) (Table 2).

**FIGURE 2:**
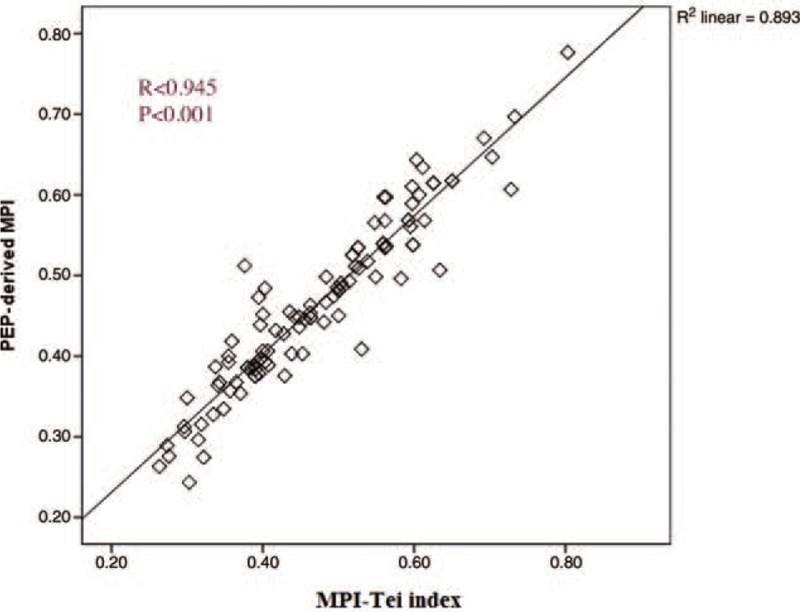
Correlation between diameter of PEP-derived MPI and MPI-Tei index in patients. MPI = myocardial performance index, MPI-Tei = myocardial performance index by using Tei method, PEP = preejection time.

PEP-derived MPI was strongly correlated with the MPI-Tei index (*r* = 0.945, *P* < 0.001) (Figure 2). In addition, PEP-derived MPI was significantly correlated with age, LVEF, E/Em, LV IVS, and LV PW (*r* = 0.429, *P* < 0.001; *r* = −0.396, *P* < 0.001; *r* = 0.292, *P* = 0.003; *r* = 0.450, *P* < 0.001; and *r* = 0.385, *P* < 0.001, respectively) (Table 3). Similarly, the MPI-Tei index was correlated with age, LVEF, E/Em, LV IVS, and LV PW (*r* = 0.443, *P* < 0.001; *r* = −0.362, *P* < 0.001; *r* = 0.327, *P* = 0.001; *r* = 0.458, *P* < 0.001; and *r* = 0.364, *P* < 0.001, respectively). The strong correlation between PEP-derived MPI and MPI-Tei index were also observed in HT group and control group, separately (*r* = 0.941, *P* < 0.001, and *r* = 0.870, *P* < 0.001, respectively).

**TABLE 3:**
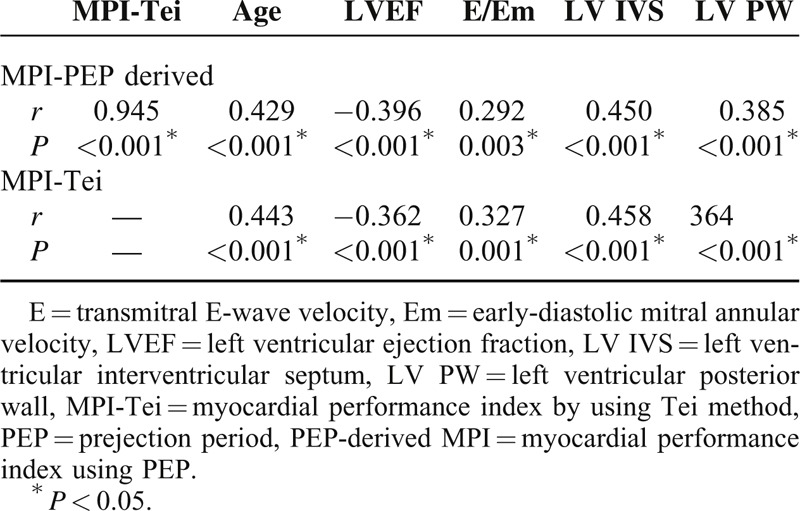
Correlation of Parameters With MPI-Tei and MPI-PEP Derived

### Reproducibility

The intraobserver mean percent errors for the PEP-derived MPI and the MPI-Tei index measurements in study patients were 4.2 ± 2.1% and 4 ± 2.1%, respectively. The interobserver mean percent errors for the PEP-derived MPI and MPI-Tei index measurements in study patients were 6.3 ± 3.2% and 5.9% ± 3.1%, respectively.

## DISCUSSION

This study revealed that the PEP-derived MPI and the MPI-Tei index were significantly higher in the HT group and there was a strong relationship between the PEP-derived MPI and the MPI-Tei index.

The MPI-Tei index is well correlated with the widely accepted systolic and diastolic hemodynamic parameters. The MPI-Tei index was determined to be a useful method in studies of congestive heart failure, congenital heart diseases, in the evaluation of interventional therapies with regard to global LV performance, in cardiac rejection following transplantation, myocardial infarction, and valvular disease.^4–7,11–21^ Hypertension causes deterioration in the systolic and diastolic function of LV.^22,23^ The relationship between HT and MPI-Tei index were investigated in a large number of studies,^24–28^ and these determined that the value of the MPI-Tei index was higher, and independently associated with LV mass index.^24–29^ Additionally, it was reported that the MPI-Tei index was related to impair systolic/diastolic parameters in echocardiography, and also determined LV deterioration in the early stages.^27–29^ In our study, we determined that mitral A wave, E/Em, IVCT, and PEP were higher, whereas E wave, S, and Em were lower in the HT group. MPI-Tei index was 0.52 ± 0.10 and the PEP-derived MPI was 0.51 ± 0.09 in the HT group, and both were significantly higher than the control group. Consistent with previously published studies, we concluded that LV systolic and diastolic function was impaired in patients with HT. PEP-derived MPI can also detect an impairment of LV systolic and diastolic function, similar to the MPI-Tei index measurement.

In this study, the MPI-Tei index was powerfully correlated with the PEP-derived MPI. In addition, both the MPI-Tei index and the PEP-derived MPI correlated with age, LVEF, E/Em, LV IVS, and LV PW. These correlations indicate that PEP-derived MPI may be useful in the evaluation of LV global function in HT.

Previous studies have utilized the PEP-derived MPI to evaluate LV function in AF patients.^8,9,29^ Recently, it was reported that the PEPa-derived MPI was a useful predictor of adverse cardiovascular events, and could offer an additional prognostic benefit over conventional clinical and echocardiographic parameters in patients with AF.^9^

We detected a strong association between the PEP-derived MPI and the MPI-Tei index. This can be explained by the following: IVRT and ET are used to determine the MPI-Tei index and the PEP-derived MPI formula. However, IVCT is used in the MPI-Tei index and PEP is used in the PEP-derived MPI. PEP and IVCT are closely related and define the preejection term.

The current estimate of AF prevalence in the developed world is approximately 1.5% to 2% of the general population. The average age of these patients is increasing steadily and averages between 75 and 85 years.^30^ The MPI-Tei index can be used in patients with sinus rhythm, but because of the loss of mechanical atrial function, cannot be utilized in patients with AF. However, PEP-derived MPI can be used in patients with both a sinus rhythm and AF. Therefore, PEP-derived MPI may be a more useful method than the Tei index. Additionally, LV function in HT patient can be evaluated using modern techniques like strain.^31–33^ The analysis of left atrial function in HT patients by novel echocardiographic techniques provides further insights into cardiac function in HT disease.^34,35^

This study has several limitations, which includes cross-sectional design, being performed at 2 different centers over a restricted time period, including a limited number of patients and absence of follow-up in terms of clinical events. We excluded patients with coronary artery disease according to medical history and ECG, but some may have been missed. We assessed LV function, but did not measure right ventricular function and invasive hemodynamic measurements in our study population. Therefore, in order to reveal the importance of PEP-derived MPI and assess its prognostic impact/role in hypertension, randomized, controlled follow-up trials involving larger groups of patients are needed.

## CONCLUSION

According to our findings, there is a strong association between the PEP-derived MPI and the MPI-Tei index. PEP-derived MPI may be used in conjunction with the MPI-Tei index, or without it, in cases of sinus rhythm to assess the LV global functions in patients with HT.
